# α-Mangostin ameliorates hepatic steatosis and insulin resistance by inhibition C-C chemokine receptor 2

**DOI:** 10.1371/journal.pone.0179204

**Published:** 2017-06-09

**Authors:** Hong Min Kim, You Mi Kim, Ji Hye Huh, Eun Soo Lee, Mi Hye Kwon, Bo Ra Lee, Hyun-Jeong Ko, Choon Hee Chung

**Affiliations:** 1 Department of Internal Medicine, Yonsei University Wonju College of Medicine, Wonju, Korea; 2 Laboratory of Microbiology and Immunology, College of Pharmacy, Kangwon National University, Chuncheon, Korea; State University of Rio de Janeiro, BRAZIL

## Abstract

Obesity induces various metabolic diseases such as dyslipidemia, nonalcoholic fatty liver disease (NAFLD), and type 2 diabetes. Fat expansion in adipose tissue induces adipose tissue dysfunction and inflammation, insulin resistance, and other metabolic syndromes. α-Mangostin (α-MG) has been previously studied for its anti-cancer, anti-inflammatory, and antioxidant activities. In this study, we investigated the effects of α-MG on adipose tissue inflammation and hepatic steatosis. We categorized study animals into four groups: regular diet control mice, RD mice treated with α-MG, high fat diet-induced obese mice, and HFD mice treated with α-MG. α-MG treatment significantly reduced not only the body, liver, and fat weights, but also plasma glucose, insulin, and triglyceride levels in HFD mice. Additionally, adiponectin levels of α-MG-treated mice were significantly higher than those of control HFD mice. Immunohistochemistry of liver and adipose tissue showed that CD11c expression was reduced in α-MG fed obese mice. α-MG treatment of HFD mice down-regulated the adipose-associated inflammatory cytokines and CCR2 in both liver and adipose tissue. Moreover, glucose tolerance and insulin sensitivity were significantly improved in α-MG fed obese mice. α-Mangostin ameliorates adipose inflammation and hepatic steatosis in HFD-induced obese mice.

## Introduction

The number of obese patients is rapidly increasing world-wide, leading to various metabolic syndromes [1–4]. Obesity is a chronic, low-grade inflammation that increases insulin resistance, reduces secretion of insulin, and increases various inflammatory reactions related to body fat [5]. It also causes metabolic syndromes such as type 2 diabetes (T2DM), non-alcoholic fatty liver disease (NAFLD), and cardiovascular disorders. If excessive accumulation of triglycerides in the liver due to obesity is prolonged, it can increase the risk of death by leading to the dysfunction of liver due to diseases such as fatty liver (steatosis), hepatocirrhosis, and hepatic fibrosis [6]. In obesity, fatty acids in the blood are increased, and excessive triglycerides accumulate in liver and adipose tissue. When liver cells malfunction, they either create excessive glucose or suppress the action of insulin, which increases blood glucose concentration by suppressing the transformation of blood glucose to glycogen [7]. Also, in the obesity, inflammation can result due to increased inflammatory cytokines (TNF-α, IL6, IL1β, etc.) and chemokines (CXCL1, CCL2, etc.) secreted from adipocytes and accumulated fat in the liver inducing insulin resistance [8]. Obesity has also been shown to induce macrophage infiltration in both rodents and human [9–11].

The activation of macrophages is known to release cytokines that induce the development of insulin resistance [12,13]. Macrophages are important immune cells that have a relationship with other immune cells (e.g., CD4 and CD8). Macrophage cell accumulation is related to interactions with chemokines and their receptors in adipose tissue [14]. The levels of chemokines, including CCL2, were increased in serum from both rodents and humans with NAFLD or NASH [15,16]. Recent studies suggest that C-C chemokine receptor 2 (CCR2) deficiency may have an influence on macrophages. CCR2 deficiency reduced the migration of macrophages in the liver and adipose tissue [17,18]. Also, our previous study demonstrated that the inhibition of CCR2 improved NAFLD and insulin resistance [19].

α-Mangostin, a type of xanthone found in mangosteen peel, has anti-inflammatory [20], antibiotic [21], anti-cancer [22], and antioxidant [23,24] effects. Also, mangosteen contains materials, such as flavonoids, anthocyanin, proanthocyanin, and phenol compounds, that react to living organisms [24]. Recently, many studies have investigated the anti-cancer and its anti-inflammatory effects of α-mangostin [25]. However, research related to its effects on diabetes has not been conducted, and its basic mechanism is unclear. This study investigated the effects of α-mangostin on insulin resistance and fatty liver in high fat diet-induced obese mice through the regulation of CCR2.

## Methods & materials

### Animal models

Six-week-old C57BL/6 mice were purchased from Koatech Laboratory Center (Gyeonggi-do, Korea) and used in all of the experiments. The mice were fed either a regular diet (RD) or a high-fat diet (HFD; 60% of calories from fat) for 12 weeks, starting at 8 weeks of age (S1 Table). The animals were maintained in an animal facility at a constant temperature of 20–22°C, 40∼60% relative humidity, and a 12 h light/12 h dark cycle for at least 7 days prior to the experiment. The mice were divided into four groups: RD-fed (control), RD-fed with α-mangostin (α-MG) treatment, HFD-fed (obese), and HFD-fed with α-MG treatment. α-Mangostin was purchased from Chengdu Biopurify Phytochemicals Ltd. (catalogue No. BP0155, China) and mixed with the feed of the treatment groups at a dose of 50mg/kg/day for 12 weeks. All extracted tissues were immediately frozen in liquid nitrogen and stored at -80°C until analysis. All experiments were conducted in accordance with the National Institutes of Health guidelines and with the approval of the Yonsei University Institutional Animal Care and Use Committee (IACUC No. YWC-131014-2).

### Glucose and insulin tolerance tests

We performed glucose tolerance tests to determine the insulin intolerance state of each group at the 16^th^ week of the study. The mice were intraperitoneally injected with insulin (0.75 U/kg body weight) after an 8-h fasting period. We performed glucose tolerance tests at the 18^th^ week of the study. The mice were intraperitoneally injected with glucose (1 g/kg body weight) after an 8-h fasting period, and blood samples were collected from the tail vein. These samples were used to measure blood glucose concentrations with a SureStep™ tester (LifeScan, Milpitas, CA, USA).

### Cell culture

RAW264.7 macrophages (ATCC, USA) were grown at 37°C and 5% CO_2_ in Roswell Park Memorial Institute (RPMI) 1640 medium (Gibco, NY, USA) containing 10% fetal bovine serum (heat-inactivated at 55°C for 30 min) and penicillin streptomycin (Invitrogen, Carlsbad, CA, USA). The medium was then replaced with RPMI 1640 containing 10% FBS and was changed every 2 days. Free fatty acids (palmitate mixture, Sigma-Aldrich) were dissolved in ethanol containing bovine serum albumin (BSA, 50 μM) and conjugated with BSA at a 10:1 molar ratio before use. Lipopolysaccharides (Sigma-Aldrich) were dissolved in ethanol and then used at 100ng/ml at 24h. Also, adipose tissue-conditioned medium (ATCM) was used at 24h.

### Isolation of adipose tissue-conditioned medium

Mesenteric adipose tissue was isolated from C57BL/6 male mice (8 weeks old; Koatech Ltd., Gyeonggi-do, Korea) fed a high-fat diet for 2 months. All subsequent procedures were performed in a laminar-flow hood. The adipose tissue was minced into fragments less than 10 mg in weight and cultured. Briefly, 500 mg of tissue was seeded in 10 ml of serum-free medium in the wells of a 100mm dish, and the dish was placed in a humidified incubator at 37°C and 5% CO_2_ for 3 days.

### Isolation of peritoneal macrophages

Mice were intraperitoneally injected with 2 ml of 3% thioglycollate. Peritoneal exudate cells were collected by lavaging the peritoneal cavity with 7 ml of RPMI 1640. The cells were centrifuged, washed, and suspended in RPMI 1640. Peritoneal macrophages were isolated from the exudate cells by adherence to tissue culture plates for 2 h.

### Hepatic triglycerides

Liver samples were weighed then homogenized in NP40. Samples were then centrifuged at 10,000 *g* for 10 min at 4°C. Triglycerides were then quantified colorimetrically as glycerol by use of a commercial enzymatic assay (Cayman Chemical, MI, USA).

### Western blot analysis

The liver of each mouse was dissected and immediately frozen in liquid nitrogen. Three hundred milligrams of each liver was homogenized with 500 μL of RIPA buffer (pH 7.4) supplemented with protease and phosphatase inhibitors. Lipids were removed by centrifugation at 10,000 × g for 20 minutes. Fifty micrograms of total protein was subjected to Western blot analysis using polyclonal antibodies to sterol regulatory element-binding transcription factor 1 (SREBP1), sterol regulatory element-binding transcription factor 2 (SREBP2), C-C chemokine receptor 2 (CCR2) phosphorylated Akt (Thr^308^), IRS-1 (Tyr^465^) or non-phosphorylated Akt, IRS-1 (Santa Cruz Biotechnology, Santa Cruz, CA, USA), and β-actin (Abcam, Cambridge, UK). All data were normalized to beta-actin, except pAkt/Akt and p-IRS1/ IRS1. And, phosphorylated Akt or IRS-1 were normalized to non-phosphorylated Akt or IRS-1. The band intensities were measured using an Image J Analyzer (Biocompare, San Francisco, CA, USA).

### Quantitative real-time PCR

Tissue and cellular RNA was extracted using TRIzol (Invitrogen), and total RNA (0.5 μg) was reverse-transcribed into cDNA according to the manufacturer’s instructions. For the quantitative real-time polymerase chain reaction (qRT-PCR) assays, the linearity of amplification of the test genes from cDNAs was established in preliminary experiments. The test genes included fatty acid synthesis genes (SREBP1c, LPL, C/EBPa, SCD1, HSL, FasN, mTOR, and PEPCK), glucose uptake genes (GLUT4, GLUT2, and IRS-1), macrophage marker (F4/80, CD11c and CD206), inflammatory cytokine genes (TNFα, IL1β, IL6, and MCP1), the anti-inflammatory cytokine gene IL10, and GAPDH (used as an internal control). cDNAs were amplified by real-time PCR in duplicate using a SYBR premix Ex Taq kit (Applied Biosystems, Foster City, CA, USA) and Thermal Cycler Dice (Life Technologies, Carlsbad, CA, USA). All reactions were performed in the same manner: 95°C for 10 seconds, followed by 45 cycles of 95°C for 15 seconds and 60°C for 1 minute. The results were analyzed using the real-time system AB 7900HT software (Life Technologies), and all values were normalized to the level of GAPDH.

### Histochemistry

Liver and adipose tissues were fixed overnight at room temperature in 4% formaldehyde and embedded in paraffin. Sections (8-μm thick) were stained with hematoxylin-eosin (H&E) and then mounted on glass slides. We quantified the fibrosis area with picrosirius red (collagen) staining using. The paraffin was then removed at 60°C for 1 hour, followed by dehydration in xylene. Polyclonal F4/80, CD11c, or CD206 antibody (Abcam, Cambridge, UK) was added in a 1:1000 dilution, and the sample was stored overnight at 4°C. The slides were evaluated using a light-microscope equipped with a charge-coupled device camera (Pulnix, Sunnyvale, CA, USA).

### Analysis of metabolic parameters

Murine blood samples were collected through cardiac puncture. Serum levels of glucose, total cholesterol, triglycerides (TG), aspartate transaminase (AST), and alanine transaminase (ALT) were determined using commercially-available enzymatic assay kits (Asan Pharmacology Co., Seoul, Korea). Serum insulin level was measured using an ultrasensitive mouse insulin ELISA kit (Shibayagi Co., Shibukawa, Japan), and adiponectin serum level was measured using an ELISA kit (AdipoGen Inc., Seoul, Korea). Both ELISA tests were performed according to the manufacturers' protocols.

### Migration assay

Cell migration was evaluated in a multi-well micro chemotaxis chamber (Neuro Probe). Peritoneal and RAW264.7 macrophages cells were suspended in either RPMI 1640, adipose tissue-conditioned medium, or CCL2 (100ng/ml) at a concentration of 1×10^4^ cells/ml, and then 60 ul of each solution was placed in the upper layer of a 96-well chamber membrane whose lower well contained or did not contain α-MG. Following incubation at 37°C for 4 h, the cells that had not migrated were removed, and the cells that migrated across the filter were fixed, stained with Diff-Quik (International Reagent Corp, Darmstadt, Germany), and counted.

### Statistical analyses

All data are presented as mean ± SEM. Statistical analysis was performed using one-way analysis of variance (ANOVA) and Tukey’s test for multiple comparisons using SPSS version 17.0 (SPSS Inc., Chicago, IL, USA). The differences were considered to be statistically significant at P < 0.05.

## Results

### α-Mangostin prevents high fat diet-induced increases in body weight

**Fig 1:**
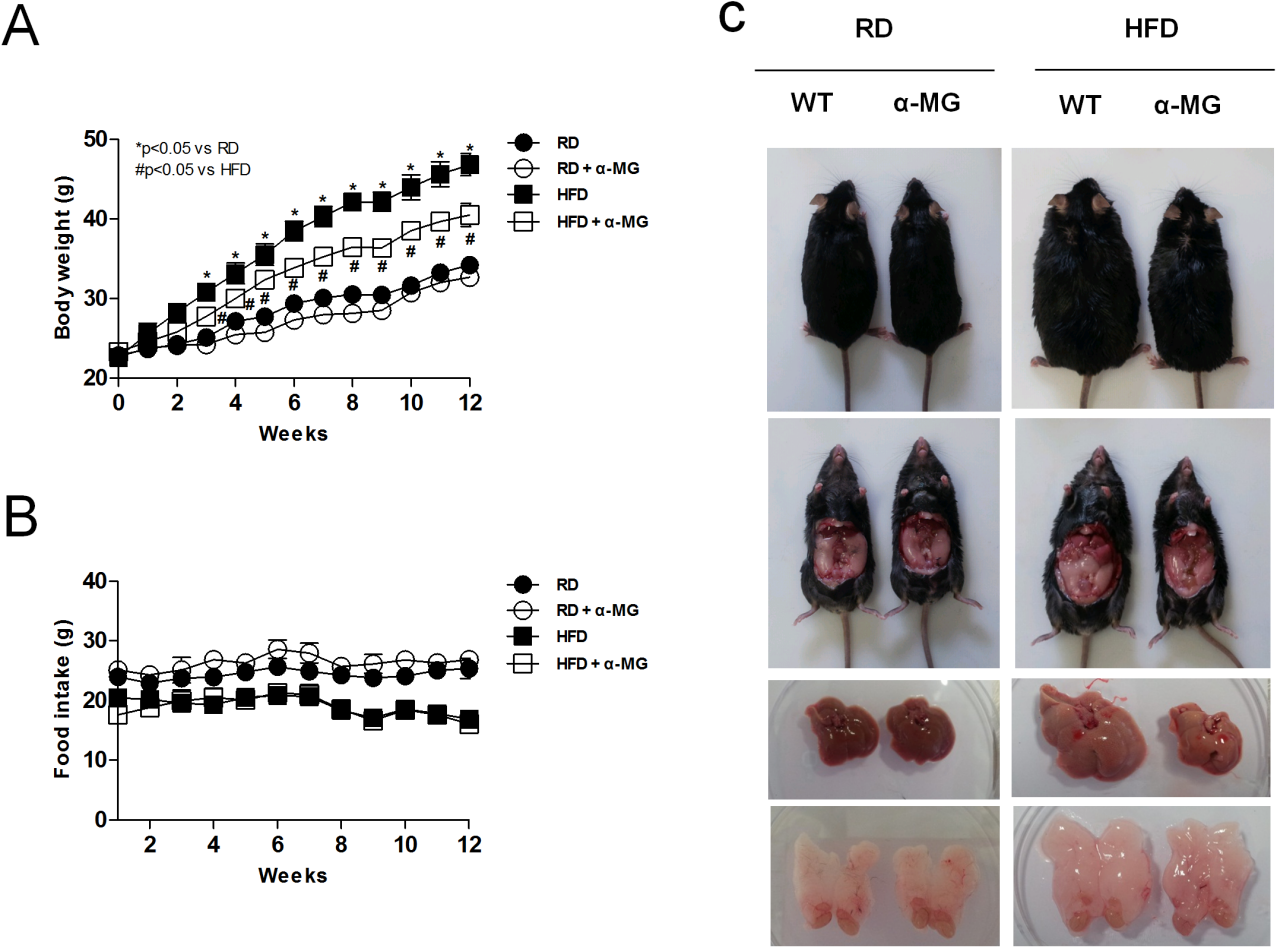
α-MG suppressed HFD-induced body weight gain in experimental animals. WT and α-MG-treated mice were fed a high-fat diet (HFD) for 12 weeks. (A) Body weight changes of WT mice (n = 10) and α-MG-treated mice (n = 10). (B) Food intake changes of WT mice and α-MG-treated mice. (C) Tissue size of WT mice and α-MG treated mice. Data are shown as mean ± SEM with n = 10 animals per group. *P < 0.05 compared with RD mice. #P < 0.05 compared to HFD mice.

To examine whether α-mangostin has an effect in diet-induced obesity, we first investigated the body weight of mice fed either a regular diet (RD) or high-fat diet (HFD), both with and without α-mangostin (α-MG), for 12 weeks. The HFD mice had significantly increased body weights compared to the RD mice, but body weights did not differ between the RD control and α-MG treated RD mice. However, the α-MG treated obese mice had significantly reduced body weights compared to the HFD mice (Fig 1A), although food intake was not different between the groups (Fig 1B). Also, tissue size was decreased in α-MG treated obese mice compared to HFD mice (Fig 1C). In the HFD mice, α-MG induced a decrease in epididymal fat and also significantly reduced the effects of fatty liver. These data suggest that α-mangostin suppresses HFD-induced weight gain.

### α-Mangostin attenuates biochemical characteristics of high fat diet-induced obese mice

To investigate the effects of α-MG on biochemical parameters, we measured the serum levels of mice that underwent regular or high-fat feeding, both with and without α-MG, for 12 weeks (Table 1). The fasting glucose and insulin levels were not different between the RD and α-MG treated RD groups. However, the levels in HFD mice were significantly higher than in α-MG treated obese mice. Total cholesterol level was significantly higher in HFD mice. Serum triglyceride level was decreased by α-MG in the RD mice. Similarly, it was significantly lower in α-MG treated obese mice than in HFD control mice. Adiponectin serum level in the α-MG treated obese mice was higher than in HFD control mice. To investigate whether α-MG could improve insulin resistance, we measured homeostatic model assessment of insulin resistance (HOMA-IR) values (Table 1). No significant differences were found between the RD and α-MG treated RD groups. However, the values were significantly increased in HFD control mice and significantly decreased in α-MG treated obese mice. These results suggest that α-MG improves insulin resistance in high fat diet-induced obese mice. In HFD mice, serum ALT level was significantly decreased by α-MG. However, no difference in serum AST level was found between HFD and α-MG treated obese mice (Table 1).

**Table 1:** Comparisons of biochemical data in control and α-MG treat mice.

	RD	RD+α-MG	HFD	HFD+α-MG
Serum glucose (mg/dl)	332.8±25.93	262.36±21.59	403.85±35.13	286.17±20.07^#^
Serum insulin (ng/ml)	1.56±0.48	1.26±0.26	5.87±1.15*	1.75±0.28^#^
Serum T-chol (mg/dl)	162±16.60	145.89±8.98	237.84±8.62*	210.82±10.70
Serum TG (mg/dl)	60.34±5.43	42.42±3.95*	86.80±3.77*	69.43±1.82^#^
HOMA-IR	0.54±1.77	0.31±0.06	2.08±0.39*	0.49±0.11^#^
Adiponectin	53.80±5.49	46.91±3.80	33.70±3.40*	48.69±2.05^#^
ALT	1.73±0.34	2.26±0.98	17.80±3.48*	3.37±0.28^#^
AST	20.96±3.73	11.29±2.42	31.35±8.17	20.59±5.07^#^

*P < 0.05 compared with RD mice.

#P < 0.05 compared to HFD mice.

### Changes in liver and white adipose tissue by α-mangostin

**Fig 2:**
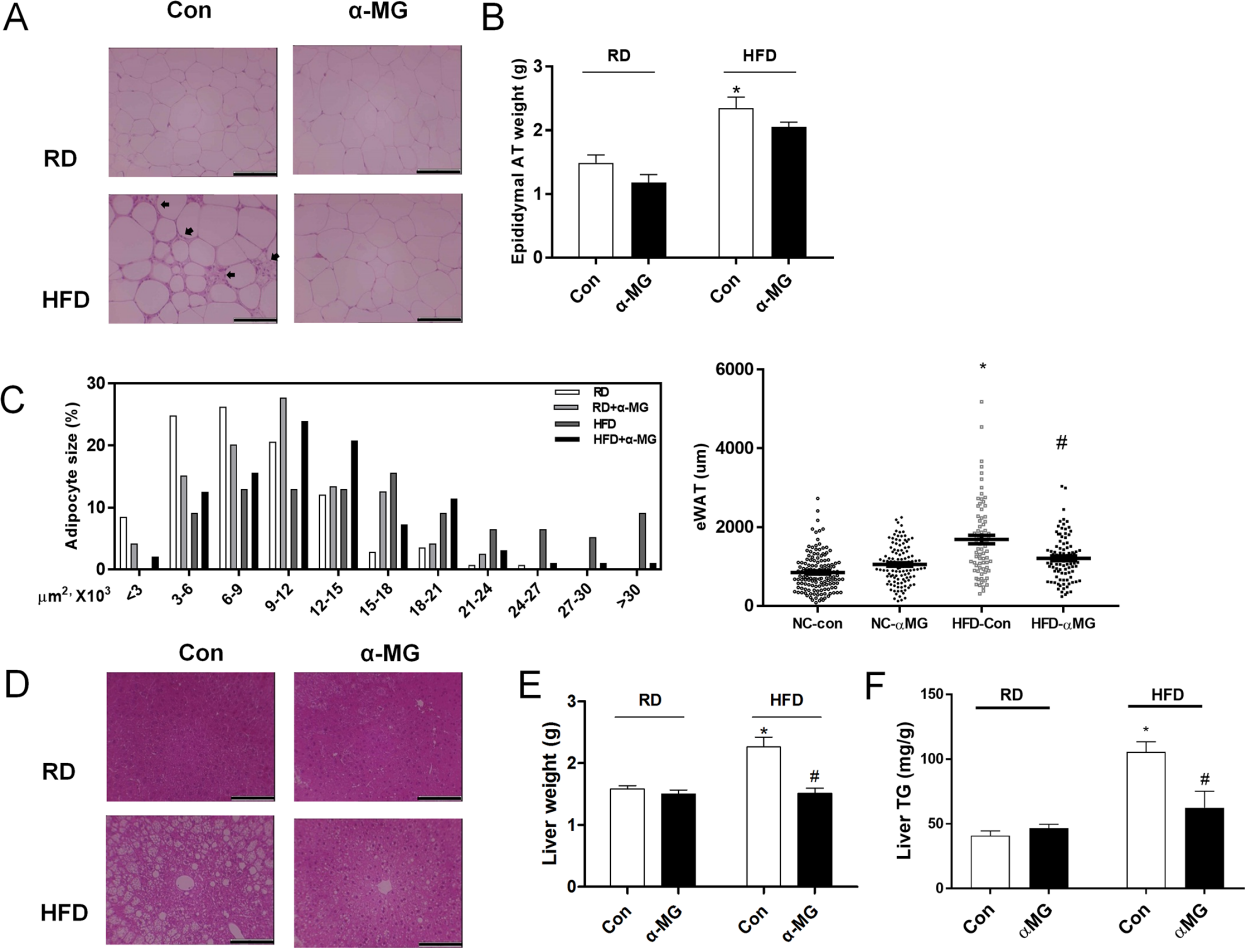
α-MG reduced liver tissue weight and epididymal adipose tissue in HFD mice. (A) Pathological grade from histological examination of the adipose tissues stained with hematoxylin and eosin. (B) Adipose tissue weight changes of WT mice and α-MG-treated mice. (C) Adipocytes size of adipose tissue in WT mice and α-MG-treated mice. (D) Pathological grade from histological examination of the livers stained with hematoxylin and eosin. (E) Liver weight changes of WT mice and α-MG-treated mice. (F) TG contents of the livers from RD- or HFD-fed WT and α-MG-treated mice. Original magnification is x200 (scale bar = 100 μm). Data are shown as mean ± SEM with n = 10 animals per group. *P < 0.05 compared to RD mice. #P < 0.05 compared to HFD mice.

Since α-mangostin suppresses HFD-induced weight gain, we measured individual tissue weights and performed histological examinations. Hematoxylin and eosin staining revealed significant expansion in the size of adipocytes in epididymal fat of HFD mice. In addition, HFD mice showed significant immune cell infiltration in the form of crown-like structures around the adipocytes. However, α-MG treated obese mice had significantly fewer crown-like structures compared to HFD mice (Fig 2A). Also, epididymal adipose tissue weight was decreased in α-MG treated obese mice compared to HFD mice (Fig 2B). Epididymal adipose tissue size was significantly reduced in α-MG treated obese mice compared to HFD mice (Fig 2C). This difference was accompanied by smaller lipid droplets in the livers of α-MG treated obese mice (Fig 2D). In addition, liver tissue weight was significantly reduced in α-MG treated obese mice compared to HFD mice (Fig 2E). The liver triglyceride levels were significantly lower in α-MG treated obese mice than in HFD mice (Fig 2F). Therefore, based on these data, α-mangostin inhibits both the expansion of adipocytes and lipid accumulation in the liver. Thus, we suggest that α-mangostin can be used to prevent high fat-diet-induced hepatic steatosis.

### α-Mangostin improves glucose tolerance and insulin sensitivity and prevents high fat diet-induced fatty acid synthesis in mice

**Fig 3:**
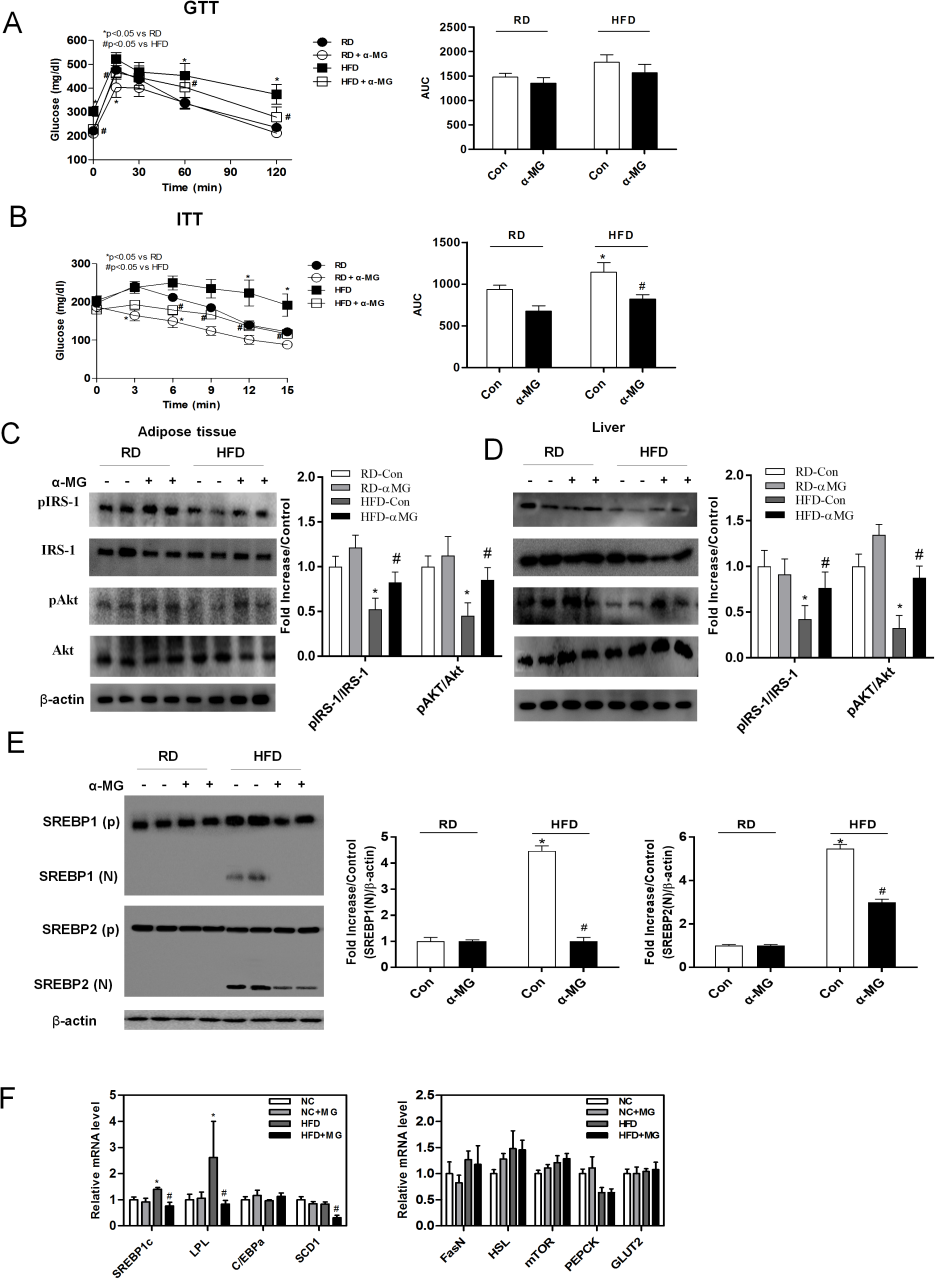
α-MG improved glucose tolerance and insulin sensitivity and reduced expression of lipid synthesis genes in the liver. (A) Mice underwent glucose (1 g/kg) tolerance tests and area under the curve of GTT. and (B) insulin (0.75 U/kg) tolerance tests and area under the curve of ITT. Western blots of insulin signaling molecules, pIRS-1 and pAkt, (C) in the adipose tissue and (D) in the liver. phosphorylated Akt or IRS-1 were normalized to non-phosphorylated Akt or IRS-1. Cropped membrane was used in western blot. (E) The expression of the proteins SREBP1(N) and SREBP2(N) was measured in liver tissue using western blots, which were re-probed for β-actin as a loading control. All data were normalized to beta-actin. The band intensities were measured using an Image J Analyzer. (F) Expression of the fatty acid synthesis genes (SREBP1c, LPL, C/EBPα, SCD1, FasN, HSL, mTOR, PEPCK and GLUT2). mRNA levels were estimated using real-time PCR. Data are shown as mean ± SEM with n = 10 animals per group. *P < 0.05 compared with RD mice. #P < 0.05 compared to HFD mice.

To examine whether α-mangostin has metabolic effects on glucose and insulin tolerance, we performed intraperitoneal glucose and insulin tolerance tests on experimental animals. During IPGTT, plasma glucose level was significantly decreased in a time-dependent manner in α-MG treated obese mice compared to HFD mice, especially at 15, 60, and 120 min (Fig 3A). Similarly, IPITT showed a stronger time-dependent decrease in α-MG treated obese mice than in HFD control mice, especially at 6, 9, 12, and 15 min (Fig 3B). To investigate the effects of α-mangostin on the regulation of insulin sensitivity, we found that levels of phosphorylated IRS-1 and phosphorylated AKT were significantly higher in the adipose tissue and in the liver of a-MG treated obese mice (Fig 3C and 3D). These data suggest that α-mangostin regulates insulin signaling in both liver and white adipose tissue. Because the TG level in liver tissue was decreased by α-MG in HFD mice (Fig 2F), we measured the expression of fatty acid synthesis genes. Western blot analysis showed reduced level of SREBP1(N) and SERBP2(N) in α-MG treated obese mice (Fig 3E). The expression of fatty acid synthesis genes (e.g., SREBP-1c, LPL, and SCD1) in liver was significantly reduced by α-MG (Fig 3F).

### α-Mangostin attenuates macrophage infiltration and inflammatory cytokines in white adipose tissue

**Fig 4:**
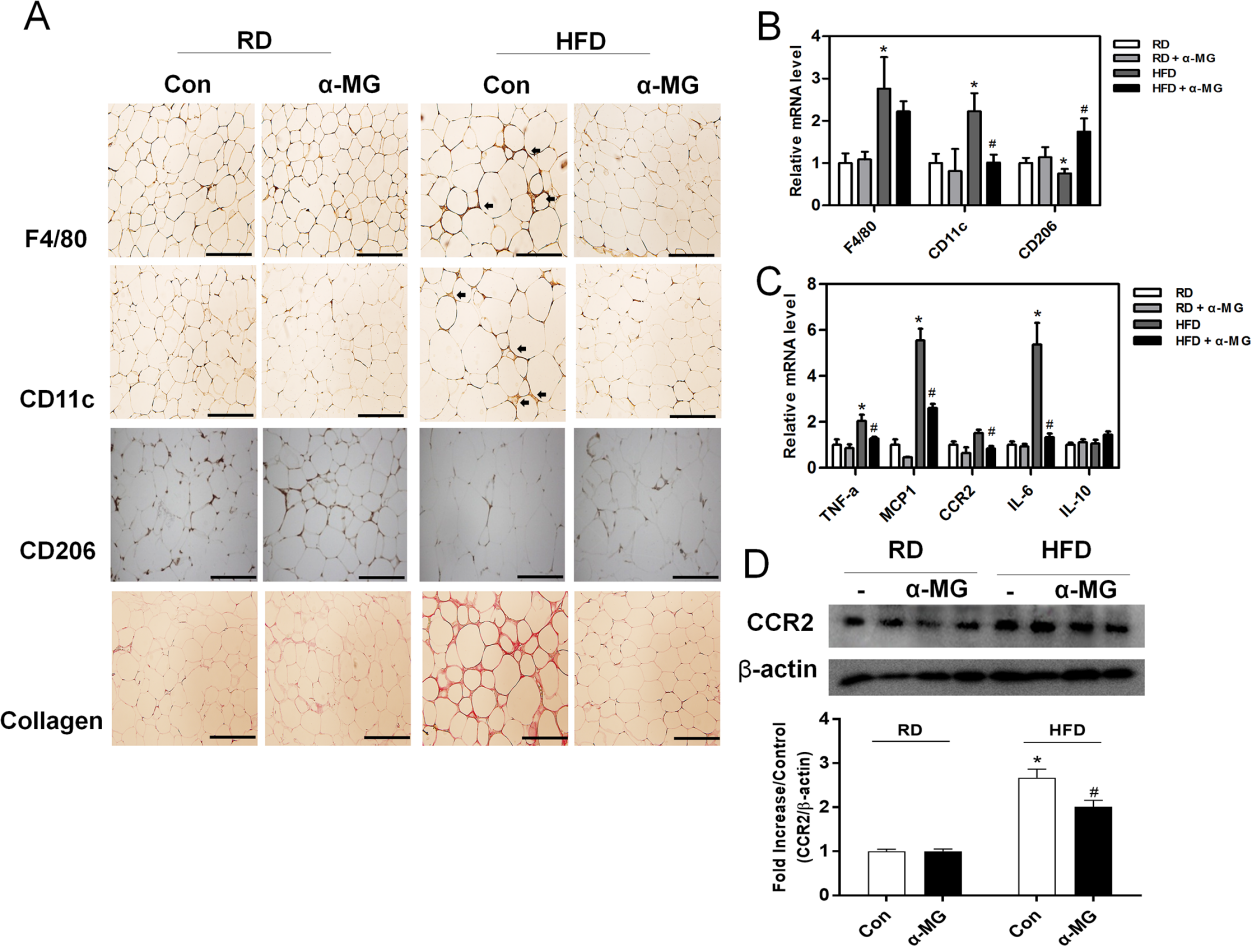
Changes in macrophages and inflammatory responses in white adipose tissue. (A) Immunohistochemistry staining of collagen and the macrophage markers F4/80 (total macrophages), CD11c (type 1 macrophages), and CD206 (type 2 macrophages) in white adipose tissue. Original magnification is x200 (scale bar = 100 μm). (B) Macrophage marker mRNA levels in white adipose tissue (n = 6). (C) Concentrations of inflammatory cytokines (TNF-α, MCP-1, CCR2 and IL-6) and the anti-inflammatory cytokine IL-10 in white adipose tissue (n = 6). mRNA levels were estimated using real-time PCR. *P < 0.05 compared to RD mice. #P < 0.05 compared to HFD mice. (D) The expression of CCR2 protein was measured in adipose tissue using western blots, which were re-probed for β-actin as a loading control. The band intensities were measured using an Image J Analyzer.

To observe whether α-MG affects high fat diet-induced inflammatory responses, we assessed the infiltration of macrophages in white adipose tissue. Macrophage infiltration was determined using specific mediators of fibrosis and inflammation. Using immunohistochemistry with the macrophage marker F4/80, we confirmed that white adipose tissue from α-MG mice showed reduced macrophage infiltration (Fig 4A). Similarly, α-MG treated obese mice had reduced level of the M1 macrophage marker CD11c, increased level of M2 macrophage marker CD206, and decreased collagen staining compared to HFD control mice (Fig 4A). The expression of macrophage genes (F4/80, CD11c, and CD206) was significantly increased in HFD mice. However, they were reduced in the white adipose tissue of HFD/α-MG mice (Fig 4B). We also examined the effects of pro-inflammatory cytokine genes (TNFα, MCP-1, CCR2, and IL-6) and anti-inflammatory cytokine gene IL-10 in white adipose tissue. Pro-inflammatory gene levels (e.g., MCP-1 and IL-6) were significantly reduced in HFD/α-MG mice. Expression of the anti-inflammatory cytokine gene IL-10 was no different among groups (Fig 4C).

### α-Mangostin attenuates macrophage infiltration and inflammatory cytokines in liver tissue

**Fig 5:**
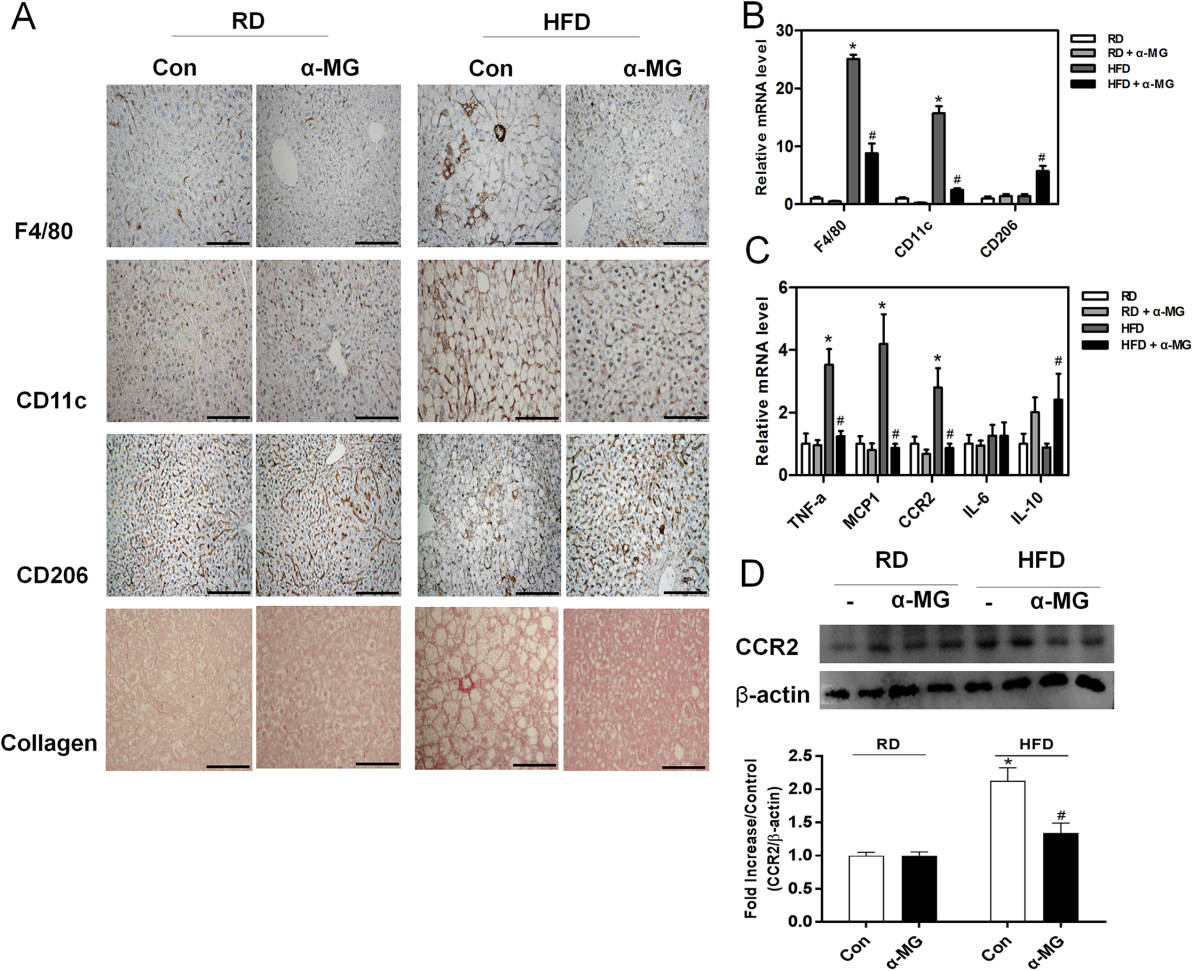
Changes in macrophages and inflammatory responses in liver tissue. (A) Immunohistochemistry staining of collagen and the macrophage markers F4/80 (total macrophages), CD11c (type 1 macrophages), and CD206 (type 2 macrophages) in liver tissue. Original magnification is x200 (scale bar = 100 μm). (B) Macrophage marker mRNA levels in liver tissue (n = 6). (C) Concentrations of inflammatory cytokines (TNF-α, MCP-1, CCR2 and IL-6) and anti-inflammatory cytokine IL-10 in liver tissue (n = 6). mRNA levels were estimated using real-time PCR. *P < 0.05 compared to RD mice. #P < 0.05 compared to HFD mice. (D) The expression of CCR2 protein was measured in liver using western blots, which were re-probed for β-actin as a loading control. The band intensities were measured using an Image J Analyzer.

Because α-MG reduced M1 macrophage and pro-inflammatory gene levels in white adipose tissue, we also examined its effects in liver tissue. Immunohistochemistry of liver tissue samples showed that α-MG reduced macrophage infiltration (F4/80), the level of M1 macrophage marker CD11c, and collagen staining compared to HFD control mice. However, α-MG increased the M2 macrophage marker CD206 in liver tissue (Fig 5A). The expression of macrophage genes (F4/80, CD11c, and CD206) was significantly reduced in α-MG treated obese mice (Fig 5B). The pro-inflammatory cytokine genes TNFα, MCP-1, and CCR2 were significantly reduced and anti-inflammatory cytokine gene IL-10 was significantly increased in α-MG treated obese mice (Fig 5C). These data suggest that α-MG decreases M1 macrophage and pro-inflammatory cytokine levels in both liver and white adipose tissue.

### α-Mangostin suppresses the migration abilities of immune cells

**Fig 6:**
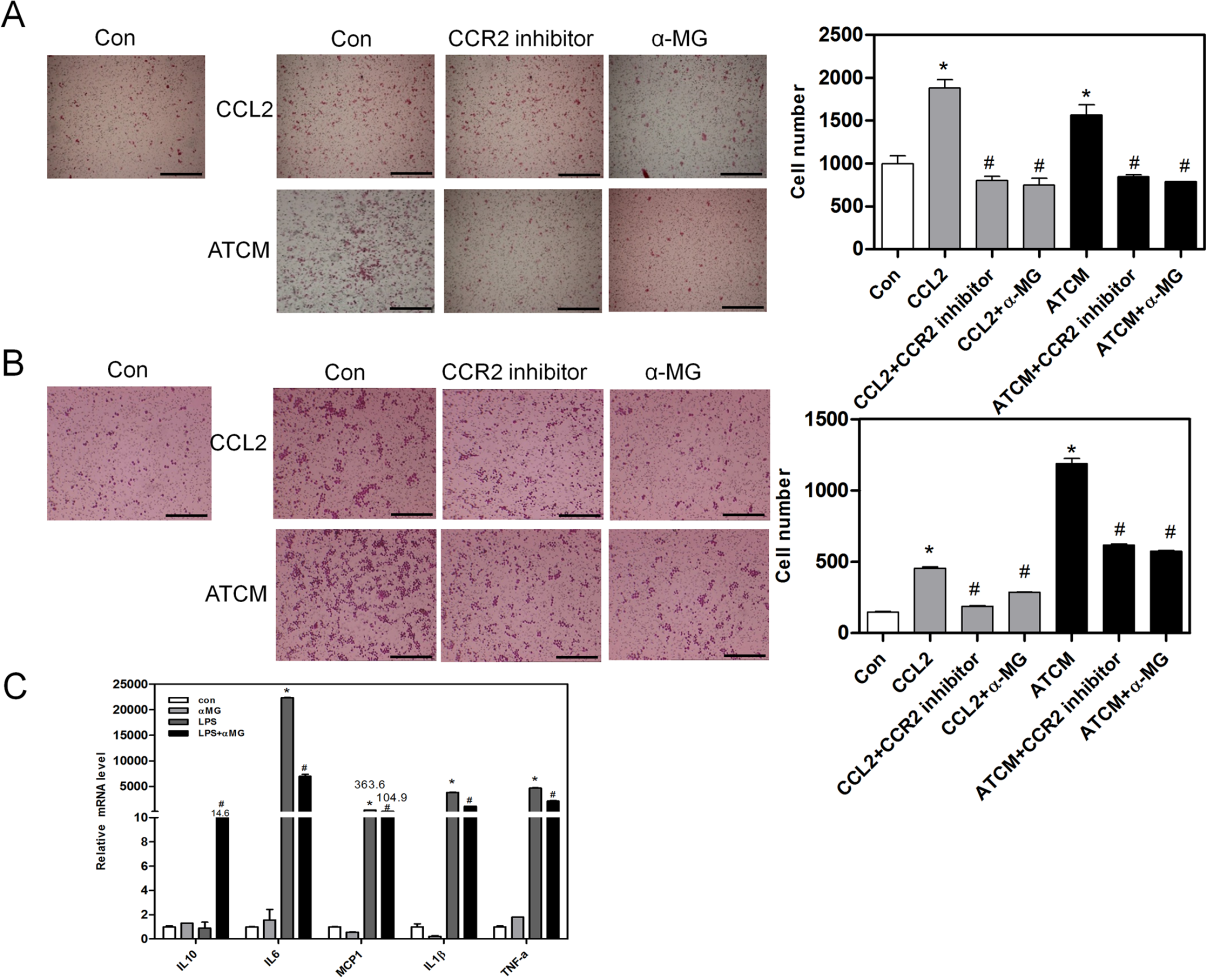
α-MG reduced inflammatory cell migration and cytokines. (A) Peritoneal macrophages and (B) Raw 264.7 (macrophage) cells were treated with CCL2 (10 ng/ml) or ATCM (adipose tissue-conditioned medium) in the presence and absence of CCR2 inhibitor (10 μM/ml) or α-MG (25 μM/ml) for 24h. The number of transmigrated peritoneal macrophages was measured using a migration assay in the presence and absence of α-MG. Original magnification is x200 (scale bar = 100 μm). (C) mRNA expression of pro-inflammatory cytokines (TNF-α, MCP-1, CCR2, and IL-6) and the anti-inflammatory cytokine IL-10 in Raw264.7 cells. mRNA levels were estimated using real-time PCR. *P < 0.05 compared to control. #P < 0.05 compared to CCL2 or ATCM treated.

To determine the role of α-MG *in vivo*, we assessed the effects of α-MG in the migration of immune cells *in vitro*. Isolated peritoneal macrophages from normal mice were treated with CCL2 and adipose tissue-conditioned media (ATCM). α-MG significantly suppressed the migration ability of peritoneal macrophages compared with CCL2-treated control (Fig 6A). This suppressive effect of α-MG was replicated in Raw264.7 cells (Fig 6B). The results indicate that α-MG can suppress the migration capacity of stimulated macrophages *in vitro*. As shown in Fig 4 and Fig 5, α-MG reduced pro-inflammatory gene levels *in vivo*. Therefore, we measured the gene levels of pro-inflammatory cytokines (TNFα, MCP-1, CCR2, and IL-6) and the anti-inflammatory cytokine IL-10 in LPS-treated Raw264.7 cells. We found that the treatment of cells with LPS increased the expression of pro-inflammatory cytokine genes, which were significantly decreased by α-MG treatment. In addition, the gene expression of anti-inflammatory cytokine IL-10 was significantly increased by α-MG treatment (Fig 6C). These data suggest that α-MG regulates macrophage migration and inflammatory-stimulated activation of CCL2, ATCM and LPS.

### α-Mangostin is not related to bone marrow differentiation into macrophages

**Fig 7:**
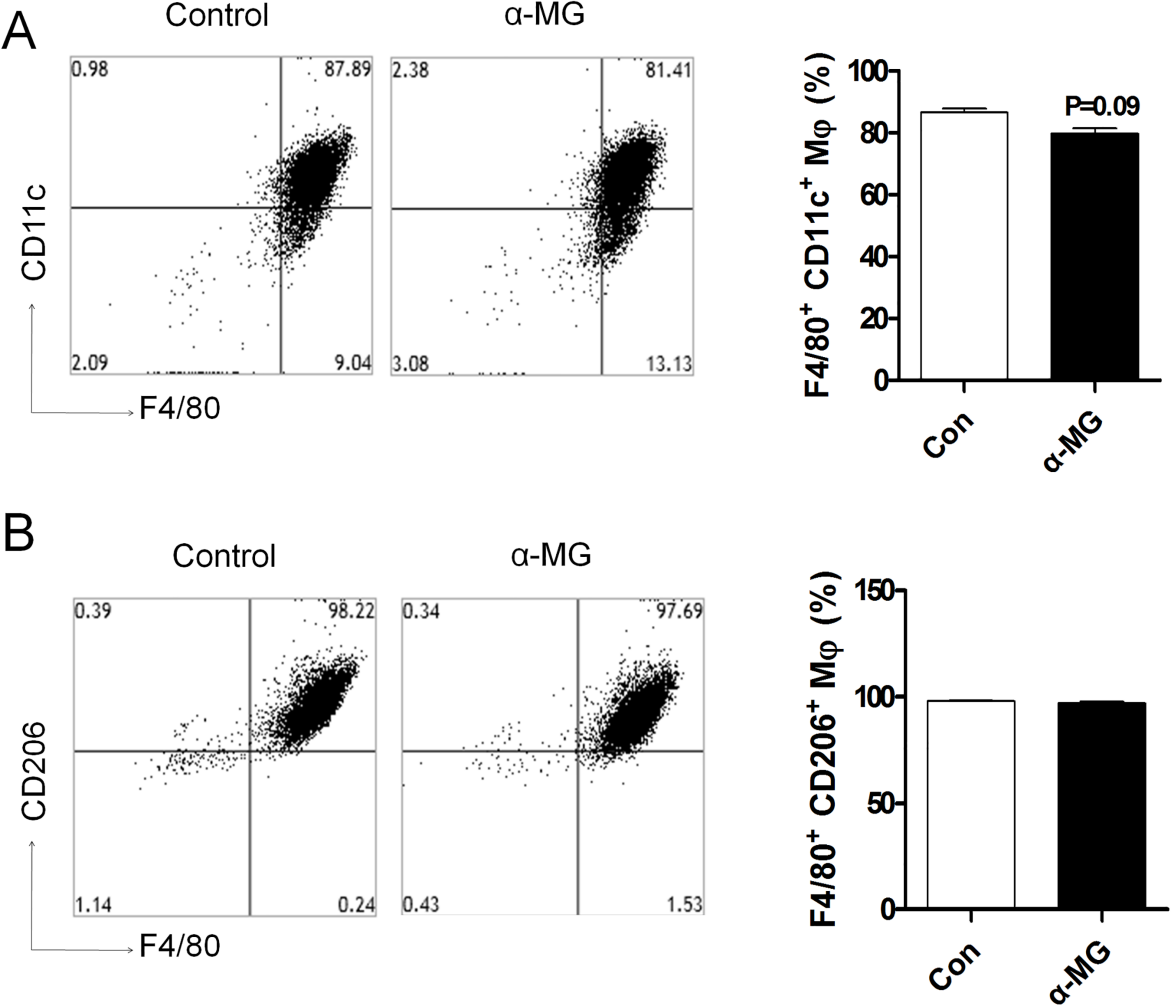
α-MG did not attenuate differentiation of bone marrow-derived macrophages. Vehicle or α-MG (10 μM/ml) were co-administrated with M-CSF (20 ng/ml) and cultured in RPMI 1640 medium containing 10% FBS and 1% penicillin at 37°C for 5 days to allow macrophage differentiation. The percentages of (A) M1 macrophages (F4/80+ CD11c+) and (B) M2 macrophages (F4/80^+^ CD206^+^) are shown. This mean was estimated by flow cytometry analysis.

To observe whether α-MG affects the differentiation of bone marrow-derived macrophages, we assessed the macrophage markers such as total macrophage (F4/80), M1 macrophage (CD11c), and M2 macrophage (CD206) using flow cytometry. We isolated bone marrow and treated it with macrophage colony-stimulating factor (M-CSF) to induce bone marrow differentiation into macrophages. Concurrent with M-CSF treatment, the samples were treated with α-MG for 5 days. Flow cytometry analysis showed that the count of M1 macrophages (F4/80^+^ CD11c^+^) (Fig 7A) was not affected by M-CSF and α-MG treatment. M2 macrophages (F4/80^+^ CD206^+^) (Fig 7B) were also not changed by α-MG treatment compared to the control. Consequently, there was no difference in the level of macrophage differentiation of bone marrow-derived cells.

## Discussion

In this study, we investigated the effects of α-MG in the liver and adipose tissue of HFD-induced obese mouse model. A hallmark of obesity is the immoderate accumulation and deposition of fat in tissues, which lead to increased triglycerides in the blood. Obesity can also lead to various metabolic syndromes and chronic diseases such as insulin resistance, hyperglycemia, cardiovascular diseases, and type 2 diabetes [26]. In this study, body weight and liver weight were significantly decreased in α-MG-treated obese mice compare to control obese mice. Additionally, adipocyte size and hepatic TG level were decreased in α-MG-treated obese mice. The present study suggests that α-MG regulates lipid homeostasis and body fat.

Several studies have shown that excessive fat accumulation in liver and adipose tissue increases inflammation. Obesity is commonly characterized by inflammation in liver and adipose tissue, which is associated with various diseases including insulin resistance, diabetes, and atherosclerosis. Inflammatory cytokines (e.g., TNFα, MCP-1, and IL-6) inhibit insulin signaling and lipid homeostasis in mice [27]. In our study, mRNA levels of inflammatory cytokines were significantly decreased in α-MG-treated obese mice, suggesting that reducing inflammation in liver and adipose tissue induces insulin signaling, which could improve the inflammation of liver and adipose tissue. Also, inducing inflammation inhibits insulin signaling pathway. We found that α-MG-treated obese mice downregulated fasting glucose and insulin levels but had significantly increased GLUT2, GLUT4, and IRS-1 mRNA levels in liver and adipose tissue. These results suggest that α-MG regulates insulin sensitivity in liver and adipose tissue.

Accumulation of macrophages induce inflammation in liver and adipose tissue. Macrophages can be divided into two types: inflammatory macrophages (M1) and anti-inflammatory macrophages (M2). Obesity is correlated to increased accumulation of M1 macrophages in liver and adipose tissue because increased level of M1 macrophages induces dysfunction and insulin resistance in liver and adipose tissue. Chen et al. demonstrated that α-mangostin has anti-inflammatory effects [20]. Our results show that obese mice have increased levels of total macrophages (F4/80) and M1 macrophages (CD11c) and decreased levels of M2 macrophages (CD206) in liver and adipose tissue. However, α-MG-treated obese mice demonstrated significant reductions in total macrophages and M1 macrophages compared to obese mice. Our data suggest that α-MG regulates macrophage migration.

Chemokines and their receptors was migrated macrophage in liver and in adipose tissue. Various chemokines, such as CCL2 and CCL19, are elevated in the serum of both humans and rodents with NAFLD [16]. CCL2-CCR2 is involved in macrophage infiltration in metabolic diseases such as obesity and diabetes [18,28]. Weisberg et al. showed that CCR2 inhibited the migration of M1 macrophages in adipose tissue [17]. Also, our previous study demonstrated that CCR2 antagonist decreased the level of M1 macrophages in liver [19]. In this study, α-MG-treated obese mice had reduced CCR2 levels in liver and in adipose tissue. We also found that α-MG had a similar effect to CCR2 inhibitors in macrophage migration. Our data suggest that α-MG inhibits CCR2, leading to the reduced migration of macrophages.

In conclusion, we demonstrated a novel effect of α-MG in liver and adipose tissue via CCR2. α-MG-treated obese mice showed decreased level of M1 macrophages in liver and adipose tissue, as well as altered insulin resistance. These results suggest that α-MG regulates macrophage-induced inflammation in liver and adipose tissue and prevents type 2 diabetes in CCR2-dependent manner.

## Supporting information

S1 Table Composition of high-fat diet (Research Diet D12492) and regular diet (Research Diet D12450J). (DOCX)
