# Supplementary material for: α-Mangostin ameliorates hepatic steatosis and insulin resistance by inhibition C-C chemokine receptor 2
Source: PLoS One. 2017 Jun 9;12(6):e0179204. doi: 10.1371/journal.pone.0179204 (PMC5466328; doi:10.1371/journal.pone.0179204)
Supplement: S1 Table — (DOCX) [file pone.0179204.s001.docx]

**S1 Table.** Composition of high-fat diet (Research Diet D12492) and regular diet (Research Diet D12450J).

| Product | D12450J | | D12492 | |
| --- | --- | --- | --- | --- |
|  | gm% | kcal% | gm% | kcal% |
| Protein | 19.2 | 20 | 26 | 20 |
| Carbohydrate | 67.3 | 70 | 26 | 20 |
| Fat | 4.3 | 10 | 35 | 60 |
| Total |  | 100 |  | 100 |
| kcal/gm | 3.85 |  | 5.24 |  |
|  |  |  |  |  |
| Ingredient | gm | kcal | gm | kcal |
| Casein, 30 Mesh | 200 | 800 | 200 | 800 |
| L-Cystine | 3 | 12 | 3 | 12 |
| Corn Starch | 506.2 | 2024.8 | 0 | 0 |
| Maltodextrin 10 | 125 | 500 | 125 | 500 |
| Sucrose | 68.8 | 275.2 | 68.8 | 275 |
| Cellulose, BW200 | 50 | 0 | 50 | 0 |
| Soybean Oil | 25 | 225 | 25 | 225 |
| Lard | 20 | 180 | 245 | 2205 |
| Mineral Mix S10026 | 10 | 0 | 10 | 0 |
| DiCalcium Phosphate | 13 | 0 | 13 | 0 |
| Calcium Carbonate | 5.5 | 0 | 5.5 | 0 |
| Potassium Citrate, 1 H2O | 16.5 | 0 | 16.5 | 0 |
| Vitamin Mix V10001 | 10 | 40 | 10 | 40 |
| Choline Bitartrate | 2 | 0 | 2 | 0 |
| Total | 1055.05 | 4057 | 773.85 | 4057 |
